# Chronic Low-Dose Alcohol Consumption Promotes Cerebral Angiogenesis in Mice

**DOI:** 10.3389/fcvm.2021.681627

**Published:** 2021-11-17

**Authors:** Jiyu Li, Chun Li, Ethyn G. Loreno, Sumitra Miriyala, Manikandan Panchatcharam, Xiaohong Lu, Hong Sun

**Affiliations:** ^1^Department of Cellular Biology and Anatomy, Louisiana State University Health Sciences Center-Shreveport, Shreveport, LA, United States; ^2^Department of Pharmacology, Toxicology and Neuroscience, Louisiana State University Health Sciences Center-Shreveport, Shreveport, LA, United States

**Keywords:** alcohol, angiogenesis, ischemic stroke, VEGF-A, VEGFR2, brain

## Abstract

Chronic alcohol consumption dose-dependently affects the incidence and prognosis of ischemic stroke. We determined the influence of chronic alcohol consumption on cerebral angiogenesis under physiological conditions and following ischemic stroke. In *in vitro* studies, acute exposure to low-concentration ethanol significantly increased angiogenic capability and upregulated vascular endothelial growth factor A (VEGF-A) and vascular endothelial growth factor receptor 2 (VEGFR2) in C57BL/6J mouse brain microvascular endothelial cells (MBMVECs). The increased angiogenic capability was abolished in the presence of a VEGFR2 inhibitor. In addition, the increased angiogenic capability and upregulated VEGF-A and VEGFR2 remained in chronically low-concentration ethanol-exposed MBMVECs. In *in vivo* studies, 8-week gavage feeding with low-dose ethanol significantly increased vessel density and vessel branches and upregulated VEGF-A and VEGFR2 in the cerebral cortex under physiological conditions. Furthermore, vessel density, vessel branches, and expression of VEGF-A and VEGFR2 in the peri-infarct cortex were significantly greater in low-dose ethanol-fed mice at 72 h of reperfusion. Although low-dose ethanol did not alter cerebral vasoreactivity and regional cerebral blood flow (rCBF) either before or during ischemia, it significantly augmented post-ischemic hyperemia during reperfusion. In contrast, exposure to high-concentration ethanol and 8-week gavage feeding with high-dose ethanol only had a mild inhibitory effect on angiogenic capability and cerebral angiogenesis, respectively. We conclude that heavy alcohol consumption may not dramatically alter cerebral angiogenesis, whereas light alcohol consumption significantly promotes cerebral angiogenesis.

## Introduction

Stroke is a leading cause of death and permanent long-term disability worldwide. Approximately 87% of all strokes are ischemic strokes, which are caused by an occlusion/narrow in an artery that supplies blood to the brain. The occlusion/narrow results in a reduction of blood flow and shortage of oxygen and nutrients, leading to injury or death of surrounding brain tissue. Thus, recanalization/reperfusion is very critical promptly for limiting ischemic brain damage. Unfortunately, recanalization/reperfusion can paradoxically induce and worsen brain damage, named reperfusion brain injury (1). Thus, promoting pre-ischemic angiogenesis may become an effective strategy to alleviate brain ischemia/reperfusion (I/R) injury by increasing collateral circulation. A recent study showed that pre-ischemic angiogenesis reduced cerebral I/R injury and improved functional recovery and survival rate in mice (2).

Angiogenesis is the generation of new blood vessels from pre-existing vessels. Angiogenesis occurs under physiological conditions as well as in several pathological processes, such as tumor growth, chronic inflammation, and ischemic stroke (3). Previous studies have shown that post-ischemic angiogenesis not only replenishes blood flow to the ischemic area of the brain but also promotes neurogenesis and improves neurological recovery in both animal models and patients (4–7). Thus, promoting post-ischemic angiogenesis may be an advantageous therapeutic approach for the treatment of ischemic stroke. Post-ischemic angiogenesis starts as early as 12–24 h and continues for more than 21 days after the onset of ischemic stroke (8). Increased vessel density can be seen in the peri-infarct area 3 days following cerebral ischemia (8). Several endogenous pro-angiogenic molecules have been identified to associate with post-ischemic angiogenesis. These molecules include vascular endothelial growth factor (VEGF), basic fibroblast growth factor (bFGF), platelet-derived growth factor (PDGF), transforming growth factor-beta (TGFβ), angiopoietin, and matrix metalloproteinases (MMPs) (9). Among these pro-angiogenic molecules, VEGF-A is a central mediator in post-ischemic angiogenesis (8). In addition, VEGF-A may have direct neuronal effects promoting neuronal survival and inducing neurogenesis (10). In the brain, many neurovascular cells including neurons, astrocytes, and vascular endothelial cells contribute to VEGF-A production and secretion (11, 12). VEGF-A binds with vascular endothelial growth factor receptor 2 (VEGFR2)/Flk-1 to initiate post-ischemic angiogenesis via PI3K/Akt, MEK/ERK, and MAPK pathways (12). On the other hand, VEGF-A and VEGFR2 also play major roles in physiological angiogenesis (13, 14).

Alcohol is one of the most commonly consumed chemical substances. The brain is a major target of alcohol actions. Increasing epidemiological and experimental studies suggest that light to moderate alcohol consumption reduces the incidence and improves the outcome of ischemic stroke, while heavy alcohol consumption increases the incidence and worsens the outcome of ischemic stroke (15–22). Recently, we found that 8-week light alcohol consumption (LAC) protected against cerebral I/R injury by suppressing post-ischemic inflammation and apoptosis and stabilizing the blood-brain barrier (BBB) in mice. On the other hand, 8-week heavy alcohol consumption (HAC) significantly worsened cerebral I/R injury by aggravating post-ischemic inflammation and BBB disruption (23, 24). Thus, our goal was to determine the influence of LAC and HAC on cerebral angiogenesis under physiological conditions and following ischemic stroke. Since we found that LAC significantly increased cerebral angiogenesis under physiological conditions, we further determined the influence of LAC on regional vasoreactivity and regional cerebral blood flow (rCBF) before, during, and after focal cerebral ischemia.

## Methods

### Mouse Brain Microvascular Endothelial Cells

To determine the influence of alcohol on angiogenic capabilities of brain endothelial cells, C57/BL6J mouse brain microvascular endothelial cells (MBMVECs) (BALB-5023; Cell Biologics) were maintained and propagated at 37°C in a humidified atmosphere of 5% CO_2_ in a CellBiologics Complete Mouse Endothelial Cell Medium that contains essential and non-essential amino acids, vitamins, organic and inorganic compounds, hormones, growth factors, trace minerals, endothelial cell growth supplement, antibiotics, and fetal bovine serum. MBMVECs at passages 4–8 were used. For studying the acute effect of alcohol, the cells were treated with 5, 10, or 50 mM ethanol either for 3 h prior to the assays or during the assays. For studying the chronic effect of alcohol, the cells were treated with 5 and 10 mM ethanol for 2 h or 50 mM for 4 h once a day for 7 days. Then the cells were processed for the following assays. To avoid the acute effect of alcohol, the last treatment of ethanol was 24 h before the assays in the study of chronic effects of alcohol.

### Matrigel Assay

24-well plates were coated with 200 μl Matrigel [BD Matrigel Growth Factor Reduced (GFR) Basement Membrane Matrix] and allowed to solidify at 37°C for 1 h. To determine the effect of acute alcohol exposure on endothelial tube formation, 400 μl MBMVECs (2.5 × 10^5^ cells/ml) were added and incubated in a medium containing 0, 5, 10, or 50 mM ethanol at 37°C for 12 h. To determine the role of VEGFR2 signaling in acute ethanol-induced tube formation, 3 μM XL184 (cabozantinib) (S1119; Selleckchem) was added into the 10 mM ethanol medium. To determine the effect of chronic alcohol exposure on endothelial tube formation, 400 μl MBMVECs (2.5 × 10^5^ cells/ml) pretreated with ethanol for 7 days were seeded and incubated in the standard medium at 37°C for 12 h. Five images (20x magnification) per well were randomly taken with a light microscope (Nikon Eclipse Ts2). The number of complete rings and total tube length per image were counted and the average was calculated to represent each well.

### Scratch Wound Healing Assay

MBMVECs were seeded in 24-well plates. After the cells reached 80–90 % confluence, a straight scratch was made per well with a 200 μl pipette tip. The wells were washed twice with fresh medium to remove the debris. To determine the effect of acute alcohol exposure on endothelial migration, the cells were incubated in a medium containing 0, 5, 10, or 50 mM ethanol at 37°C for 24 h. To determine the role of VEGFR2 signaling in acute ethanol-induced migration, 3 μM XL184 was added into the 10 mM ethanol medium. To determine the effect of chronic alcohol exposure on endothelial migration, 7-day ethanol-pretreated cells were incubated in the standard medium at 37°C for 24 h. Five images (20x magnification) per well over the gap area were randomly taken with a light microscope (Nikon Eclipse Ts2) immediately and 24 h after scratching. The changes in the gap areas were measured using ImageJ (NIH) and expressed as percentage changes to the control.

### Proliferation Assay

To determine the effect of acute alcohol exposure on the proliferation of MBMVECs, 100 μl MBMVECs (2.5 × 10^5^ cells/ml) pretreated with 5, 10, or 50 mM ethanol for 3 h were added into 96-well plates and incubated at 37°C for 4 h. To determine the role of VEGFR2 signaling in acute ethanol-induced proliferation, 3 μM XL184 was added into the 10 mM ethanol medium. To determine the effect of chronic alcohol exposure on the proliferation of MBMVECs, 7-day ethanol-pretreated cells were seeded and incubated at 37°C for 4 h. Subsequently, 10 μl of Cell Counting Kit-8 (CCK-8, Dojindo Molecular Technologies) reagent was added into each well and the cells were further incubated for 2 h. The absorbance at 450 nm was measured with the FLUOstar Omega microplate reader (BMG LABTECH) and expressed as percentage changes to the control.

### Animal Models

All of the procedures and protocols were approved by the Institutional Animal Care and Use Committee (IACUC) at the Louisiana State University Health Science Center (LSUHSC)-Shreveport and performed following the National Institutes of Health *Guide for the Care and Use Laboratory Animals* and the ARRIVE *(Animal Research: Reporting in vivo Experiments)* guidelines. Sixty-two male C57BL/6J mice (3 months) (25–30 g) were randomly divided into 3 groups and gavage fed with 0.7 g/kg (designated as LAC, *n* = 22), 2.8 g/kg (designated as HAC, *n* = 18) ethanol, or volume-matched water (designated as control, *n* = 22) once a day for 8 weeks. At the end of 8 weeks of feeding, body weight, mean arterial blood pressure (MABP), heart rate, and fasting glucose level were measured. Nine mice from each group were euthanized to measure vessel density of the brain and expression of VEGF-A and VEGFR2. Nine mice from each group were subjected to transient focal cerebral ischemia for measuring post-ischemic angiogenesis and expression of VEGF-A and VEGFR2. Eight mice from the LAC group and control group were subjected to transient focal cerebral ischemia for measuring vascular response and regional cerebral blood flow (rCBF) during ischemia and early reperfusion.

### Transient Focal Cerebral Ischemia

Transient focal cerebral ischemia was induced by unilateral middle cerebral artery occlusion (MCAO) for 60 min as described previously (23). To avoid a possible effect of acute ethanol, ethanol was not given on the day before the experiment. The mouse was anesthetized with isoflurane (induction at 5% and maintenance at 1.5%) in a gas mixture containing 30% O_2_/70% N_2_. The body temperature was maintained with a temperature-controlled heating pad (Harvard Apparatus, March, Germany) during the procedure, rCBF of the right MCA territory was monitored by a Laser-Doppler flow probe (PERIMED, PF 5010 LDPM Unit, Sweden). To occlude the right MCA, the right common carotid artery (CCA) and external carotid artery (ECA) were exposed and ligated. Subsequently, a silicon rubber-coated monofilament (Doccol Corporation, Sharon, MA, USA) was inserted from the basal part of the right ECA and advanced cranially into the right internal carotid artery (ICA) to the point where the ICA bifurcates into the MCA and the anterior cerebral artery (ACA). A rapid drop in rCBF indicated the onset of MCAO. After a 60-min of occlusion, the monofilament was removed and the CCA was reopened to achieve reperfusion. Mice were euthanized at 72 h of reperfusion to measure post-ischemic angiogenesis and expression of VEGF-A and VEGFR2 in the brain.

### Western Blot Analysis

To measure the expression of VEGF-A and VEGFR2, 3-h and 7-day ethanol-treated MBMVECs, cerebral cortex, and the peri-infarct cortex were homogenized in ice-cold lysis buffer (10 mM EDTA, 0.1% Tween-20, 1%Triton, 0.1% mercaptoethanol, 150 mM NaCl, 50 mM Tris HCl, 5 μg/ml leupeptin, 0.1 mM phenylmethylsulfonyl fluoride, and 5 μg/ml aprotinin, pH 7.4). Homogenates were centrifuged at 12,000 RPM for 20 min at 4°C. The protein concentration of the supernatants was measured with the Bradford method (Bio-Rad, CA, USA). SDS polyacrylamide gel electrophoresis (SDS-PAGE) was performed on a 10% gel. Following SDS-PAGE, the proteins were transferred onto the polyvinylidene difluoride membrane. Immunoblotting was performed using rabbit anti-VEGF-A (ab46154; Abcam), rabbit anti-VEGFR2 (2479s; Cell Signaling), and mouse anti-GAPDH (sc-32233; Santa Cruz) as primary antibodies and Peroxidase-AffiniPure Goat Anti-Rabbit IgG (111-035-144; Jackson) and anti-mouse IgG HRP-linked antibody (7076S; Cell Signaling) as the secondary antibodies. The target proteins were subsequently detected with the enhanced chemiluminescence (ECL) kit (Pierce Chemical, IL). The band densities were analyzed using ChemiDoc™ MP Imaging System (Bio-Rad). For quantification, protein expression of VEGF-A and VEGFR2 was normalized to GAPDH and expressed as percentage changes to the control.

### Immunohistochemistry Staining

To determine the effect of chronic alcohol consumption on cerebral angiogenesis, mice were anesthetized and perfused transcardially with phosphate-buffered saline (PBS), followed by 4% paraformaldehyde. The brains were removed, fixed overnight in 4% paraformaldehyde, dehydrated in a graded series of sugar solutions for 72 h, then embedded in O.C.T. compound (Fisher Scientific), and quickly frozen for 5 min in liquid nitrogen. The frozen brains were cut into 14 μm coronal sections and placed on frost-free slides. Three sections (1.21 mm rostral and 0.23 and 1.31 mm caudal to bregma) from each mouse were washed with PBS, blocked with a mixture of 10% bovine serum albumin (BSA) and 0.1% Trypsin for 1 h, and subsequently incubated with 1:100 goat anti-CD31 antibody (AF3628; R&D Systems) overnight at 4°C. Following washes, the sections were incubated with 1:200 biotinylated rabbit anti-goat lgG antibody (BA-5000; Vector Labs) for 1 h, then incubated with 1:200 streptavidin Alexa Fluor™ 488 conjugate (s32354; Thermo Fisher) for 30 min at room temperature. Following washes, sections were covered with DAPI mounting medium (VectorShield) and observed using a fluorescence microscope (Nikon Eclipse Ts2). For quantitative analysis, four images were taken from each region of interest. Vessel density and the number of branches were quantified using NIS-Elements software (Nikon) and expressed as percentage changes to the control.

### Measuring Regional Vasoreactivity and rCBF

Eight mice from LAC and control groups were anesthetized with thiobutabarbital sodium (Inactin, 100 mg/kg, ip), and a tracheotomy was performed. The mice were ventilated mechanically with room air and supplemental oxygen using a small animal ventilator (Harvard apparatus, March, Germany) at a fixed inhalation-exhalation ratio (1:1). Blood pressure was measured using a CODA mouse tail-cuff system (Kent Scientific, Torrington, CT, USA). Body temperature was maintained at 37°C using a rectal temperature-regulated heating pad (TC1000, CWE, Ardmore, PA, USA). To perform ischemia/reperfusion and observe regional vasoreactivity and rCBF, the mice were placed on a stereotaxic frame. A cranial window (6 mm × 8 mm, 1 mm from the midline to the zygomatic arch) was prepared over the left frontal, parietal and temporal cortex. The cranial window was suffused with artificial cerebrospinal fluid (CSF) bubbled continuously with 95% N_2_ and 5% CO_2_. The temperature of the CSF was maintained at 37°C. This method will maintain the temperature, pH, PCO_2_, and PO_2_ of the cranial window at a normal physiological range during the experiment. As described previously, the MCA was ligated at its M2 segment just proximal to the first bifurcation/trifurcation with a 10-0 nylon suture for 2 h (25). To prevent potential damage to the MCA during making and releasing the ligation, a segment of 5-0 monofilament nylon suture was ligated together with the MCA. Removing the nylon suture at 2 h of ischemia-induced reperfusion. Image recording of cerebral vasculature was started 1 min before MCA ligation and continued throughout the experiment with an Orca-Flash 2.8 CMOS camera (Szxz-FOF, Olympus, Japan). The images at 5, 10, 30, 60, 90, and 120 min of ischemia and 5, 10, 30, 60, 90, 120, 150, and 180 min of reperfusion were analyzed with a Visiopharm Integrator System (Olympus, Japan). Percentage change of vascular diameter at the middle of the MCA branches, arterial anastomoses, and terminal branches of the anterior cerebral artery (ACA)/posterior cerebral artery (PCA), which are anastomosing the terminal branches of the MCA, were calculated. In addition, the rCBF of four regions of interest (ROIs) from the proximal to the distal to the site of ligation was measured using Laser Speckle Contrast Imaging System (PeriCamPSI system, Perimed, Stockholm, Sweden) at the time points when cerebral vasculature was imaged. The rCBF in sequential order from the ligation was analyzed and expressed as percentage changes to the baseline.

### Statistical Analysis

All the quantitative data are presented as means ± standard deviation (SD). Prism 9 was used for statistical analyses. One-way ANOVA followed by Dunnett's test was performed for multiple comparisons to the control group. An unpaired *t*-test was performed for the comparison of two independent groups. The differences are considered statistically significant as *P*-value < 0.05.

## Results

### Effect of Acute Alcohol Exposure on Angiogenic Capability of MBMVECs

**Figure 1:**
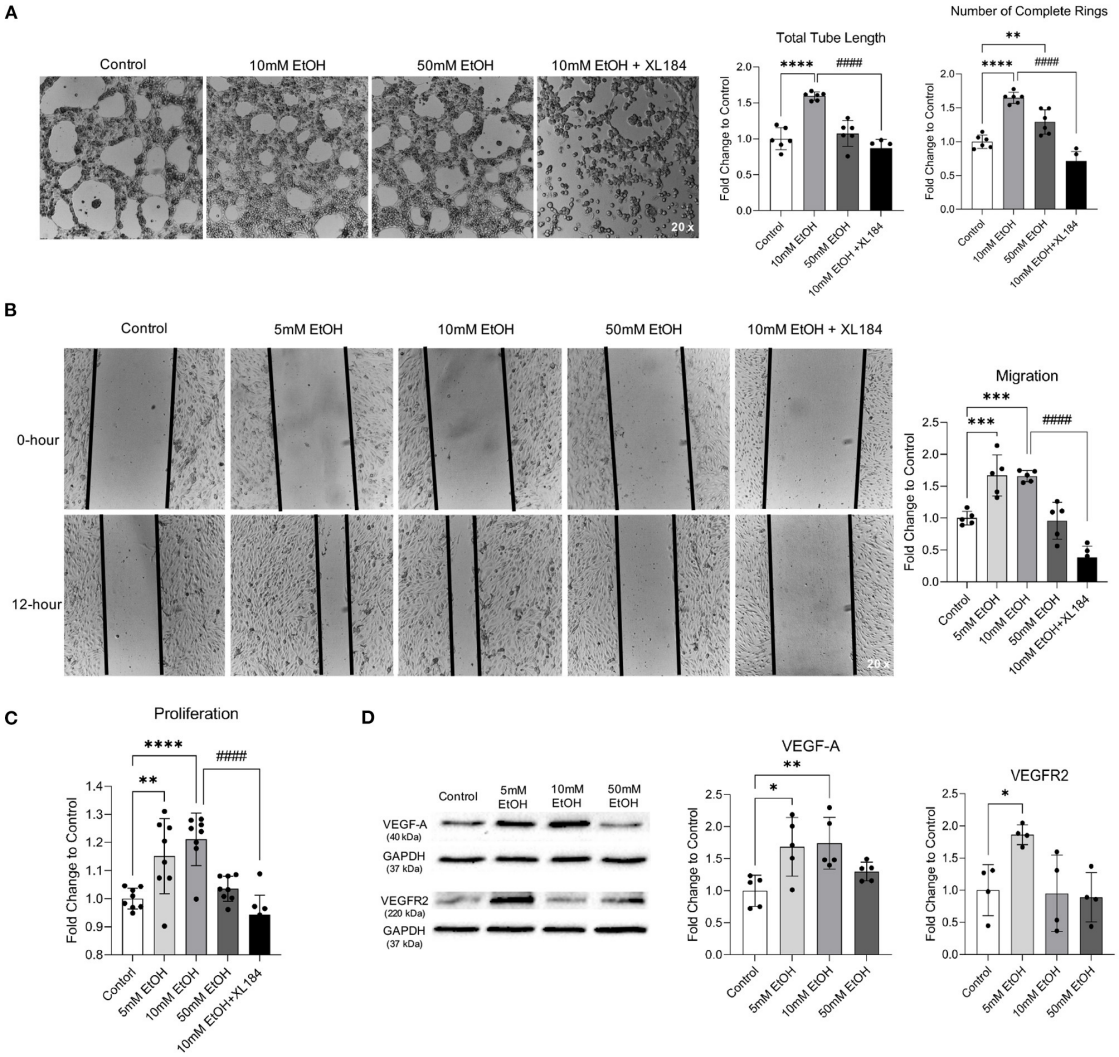
Effect of acute alcohol exposure on tube formation, migration, proliferation, and expression of VEGF-A and VEGFR2 in MBMVECs. **(A)** Representative images of tube formation (left). Values are means ± SD (right, *n* = 6). **(B)** Representative images (left) of migration. Values are means ± SD (right, *n* = 5). **(C)** Values are means ± SD (*n* = 8) for proliferation. **(D)** Representative Western blots of VEGF-A and VEGFR2 (left). Values are means ± SD (right, *n* = 4–5). ^*^P < 0.05, ^**^P < 0.005, ^***^P < 0.0005, ^****^P < 0.0001 vs. Control. ####*P* < 0.0001 vs. 10 mM EtOH. Analyzed using one-way ANOVA with Dunnett's *post-hoc*.

Acute exposure to low concentration (5 and 10 mM) ethanol significantly increased the total tube length and number of complete rings in Matrigel Assay, migration in Scratch Wound Healing Assay, and proliferation in Proliferation Assay (Figures 1A–C). In addition, 5 mM ethanol significantly upregulated both VEGF-A and VEGFR2 and 10 mM ethanol significantly upregulated VEGF-A (Figure 1D). Furthermore, VEGFR2 inhibitor, XL184, abolished 10 mM ethanol-induced tube formation, migration, and proliferation (Figures 1A–C). In contrast, acute exposure to 50 mM ethanol did not alter the migration, proliferation, and expression of VEGF-A and VEGFR2 (Figures 1B–D). But it significantly increased the number of complete rings in Matrigel Assay (Figure 1A).

### Effect of Chronic Alcohol Exposure on Angiogenic Capability of MBMVECs

**Figure 2:**
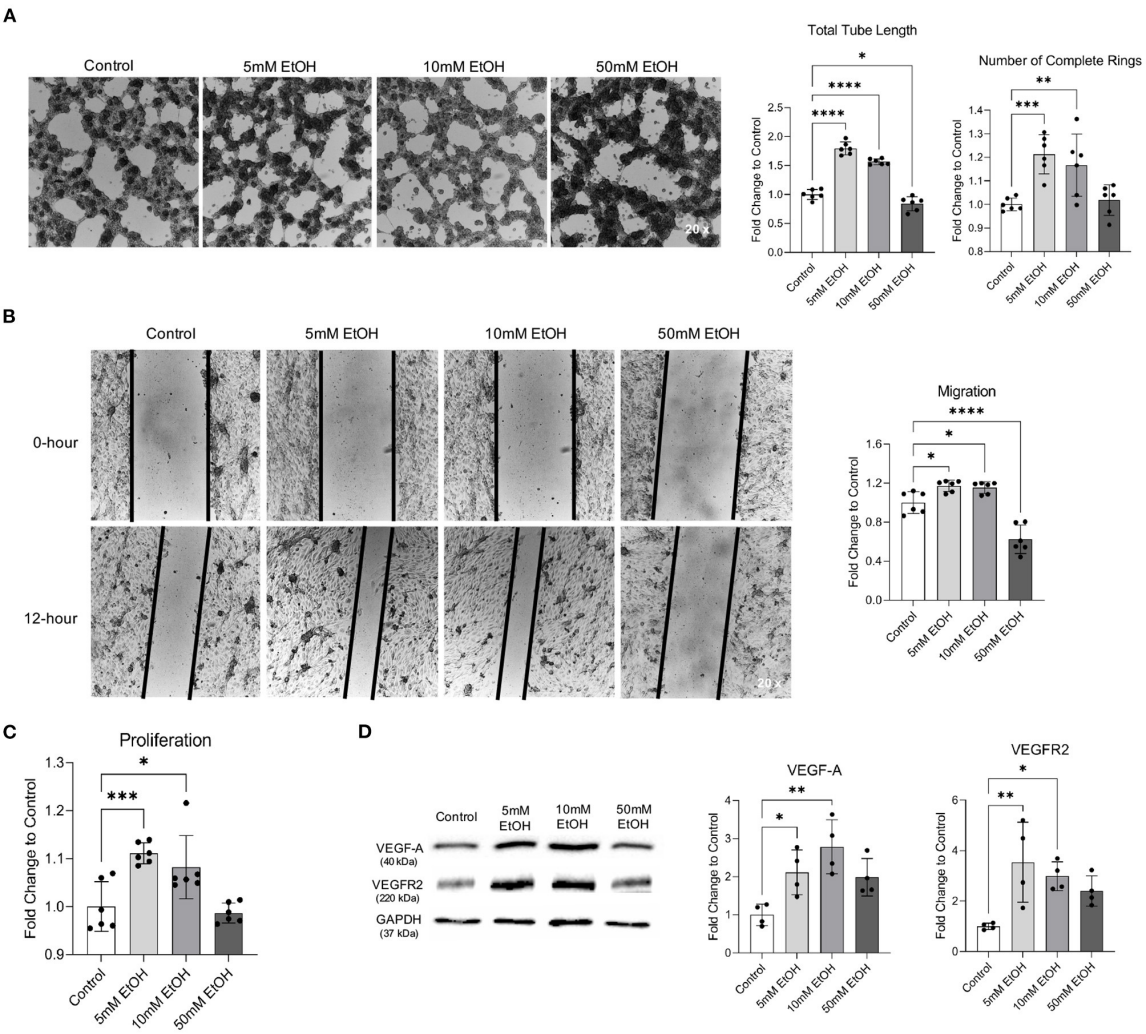
Effect of chronic alcohol exposure on tube formation, migration, proliferation, and expression of VEGF-A and VEGFR2 in MBMVECs. **(A)** Representative images of tube formation (left). Values are means ± SD (right, *n* = 6). **(B)** Representative images (left) of migration. Values are means ± SD (right, *n* = 6). **(C)** Values are means ± SD (*n* = 6) for proliferation. **(D)** Representative Western blots of VEGF-A and VEGFR2 (left). Values are means ± SD (right, *n* = 4). ^*^P < 0.05, ^**^P < 0.005, ^***^P < 0.0005, ^****^P < 0.0001 vs. Control. Analyzed using one-way ANOVA with Dunnett's *post-hoc*.

Two-hour exposure to 5 and 10 mM ethanol once a day for 7 days significantly increased the total tube length and number of complete rings in Matrigel Assay, migration in Scratch Wound Healing Assay, and proliferation in Proliferation Assay (Figures 2A–C). In addition, 7-day exposure to 5 and 10 mM ethanol significantly upregulated both VEGF-A and VEGFR2 (Figure 2D). In contrast, although 4-h exposure to 50 mM ethanol once a day for 7 days did not alter the number of complete rings in Matrigel Assay, proliferation in Proliferation Assay, and expression of VEFG-A and VEGFR2 (Figures 2A,C,D), it significantly reduced the total tube length in Matrigel Assay and the migration in Scratch Wound Healing Assay (Figures 2A,B).

### Effect of Chronic Alcohol Consumption on Cerebral Angiogenesis Under Physiological Conditions

**Figure 3:**
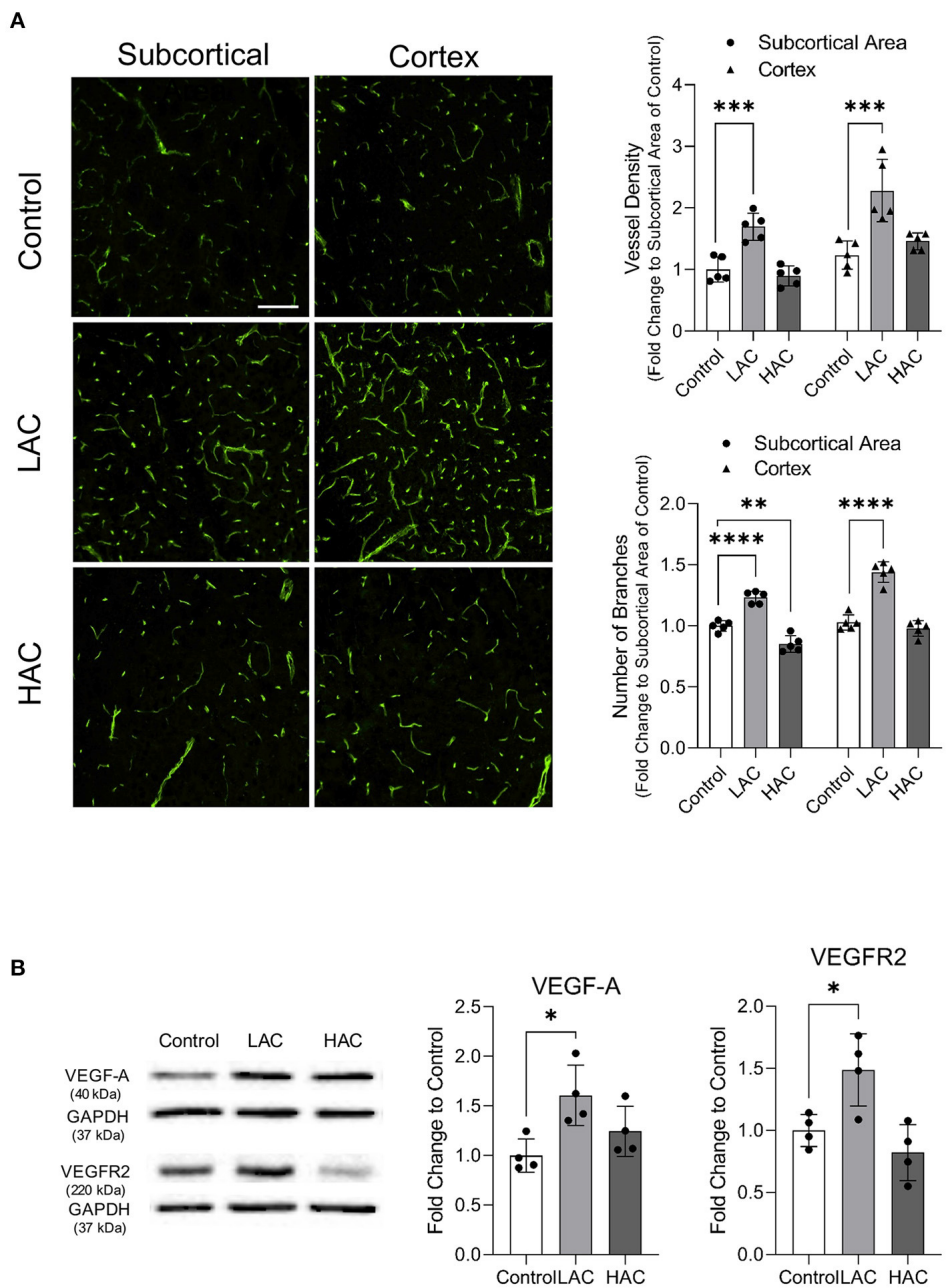
Effect of chronic alcohol consumption on cerebral angiogenesis under physiological conditions. **(A)** Representative images of CD31 staining (left). Values are means ± SD (right, *n* = 5). **(B)** Representative Western blots of VEGF-A and VEGFR2 (left) (scale bar = 100 μm). Values are means ± SD (right, *n* = 4). ^*^P < 0.05, ^**^P < 0.005, ^***^P < 0.0005, ^****^P < 0.0001 vs. Control. Analyzed using one-way ANOVA with Dunnett's *post-hoc*.

As reported previously, daily gavage feeding with either 0.7 or 2.8 g/kg/day ethanol once a day for 8 weeks did not significantly change body weight, MABP, heart rate, and fasting glucose level (23, 24) (data not shown). However, 8-week feeding with 0.7 g/kg/day ethanol significantly increased vessel density and the number of branches in the cerebral cortex and subcortical area under physiological conditions (Figure 3A). Consistently, 0.7 g/kg/day ethanol feeding significantly upregulated VEGF-A and VEGFR2 in the cerebral cortex (Figure 3B). In contrast, 8-week feeding with 2.8 g/kg/day ethanol did not alter either vessel density or the number of branches in the cerebral cortex under physiological conditions (Figure 3A). It only slightly but significantly reduced the number of branches in the subcortical area. However, 2.8 g/kg/day ethanol feeding did not significantly alter either VEGF-A or VEGFR2 in the cerebral cortex (Figure 3B).

### Effect of Chronic Alcohol Consumption on Cerebral Angiogenesis Following Ischemia/Reperfusion

**Figure 4:**
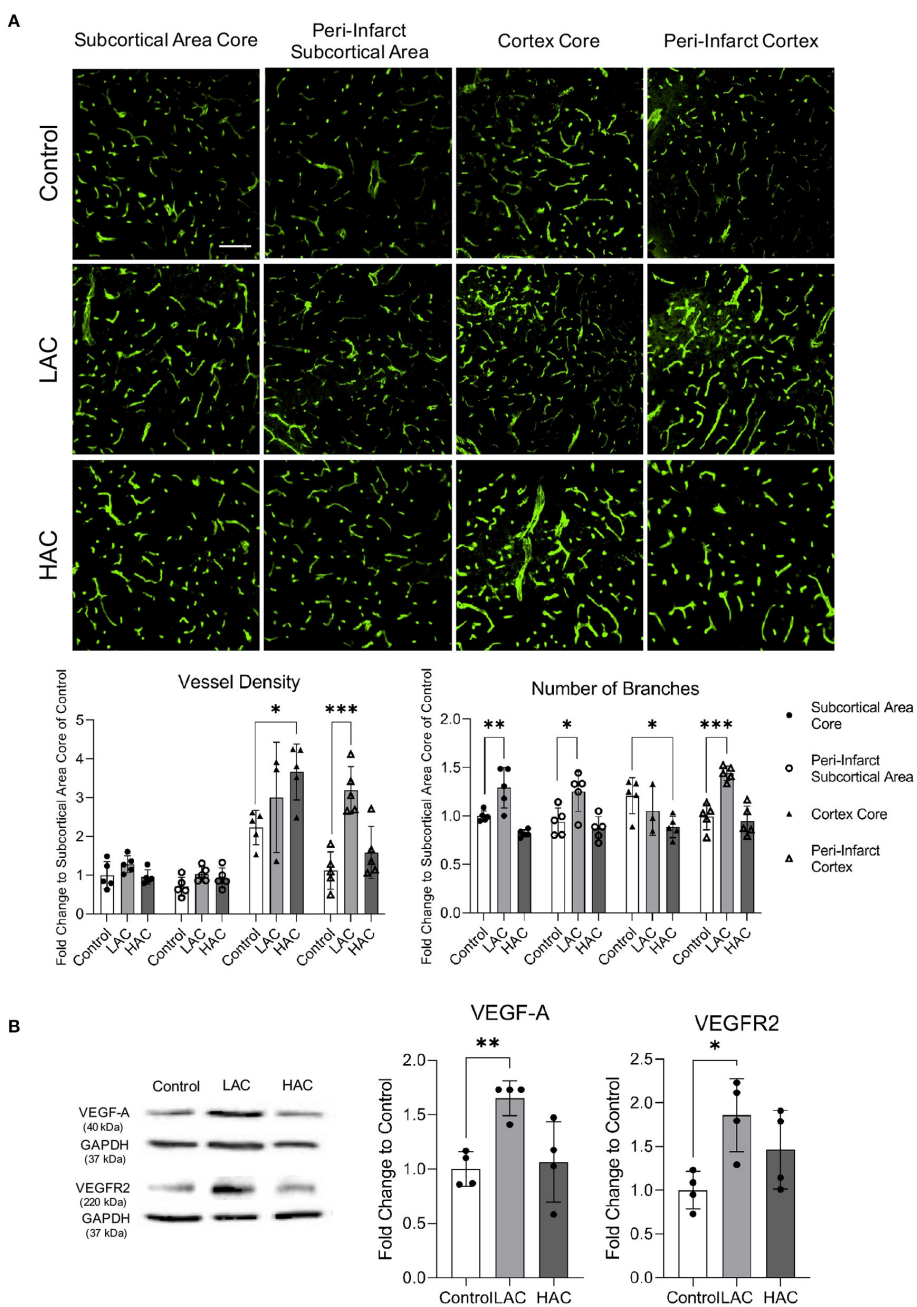
Effect of chronic alcohol consumption on cerebral angiogenesis at 72 h of reperfusion following a 60-min transient focal cerebral ischemia. **(A)** Representative images of CD31 staining (upper) (scale bar = 100 μm). Values are means ± SD (lower, *n* = 5). **(B)** Representative Western blots of VEGF-A and VEGFR2 (left). Values are means ± SD (right, *n* = 4). ^*^P < 0.05, ^**^P < 0.005, ^***^P < 0.0005 vs. Control. Analyzed using one-way ANOVA with Dunnett's *post-hoc*.

Cerebral ischemia produced cerebral angiogenesis in the ischemic core and peri-infarct area. Vessel density and number of branches at 72 h of reperfusion in the peri-infarct cortex were significantly greater in 0.7 g/kg/day ethanol-fed mice compared to the control mice (Figure 4A). Consistently, protein expression of VEGF-A and VEGFR2 in the peri-infarct cortex was significantly greater in 0.7 g/kg/day ethanol-fed mice compared to the control mice (Figure 4B). In addition, 0.7 g/kg/day ethanol feeding significantly increased the number of branches in the ischemic core and peri-infarct subcortical area (Figure 4A). On the other hand, 2.8 g/kg/day ethanol feeding significantly increased vessel density in the ischemic core of the cerebral cortex. However, it significantly reduced the number of branches in the ischemic core of both the cerebral cortex and subcortical area (Figure 4A). In addition, 2.8 g/kg/day ethanol feeding did not alter the post-ischemic expression of either VEGF-A or VEGFR2 in the peri-infarct cortex at 72 h of reperfusion (Figure 4B).

### Effect of Chronic Alcohol Consumption on Regional Vasoreactivity and rCBF During Ischemia and Reperfusion

**Figure 5:**
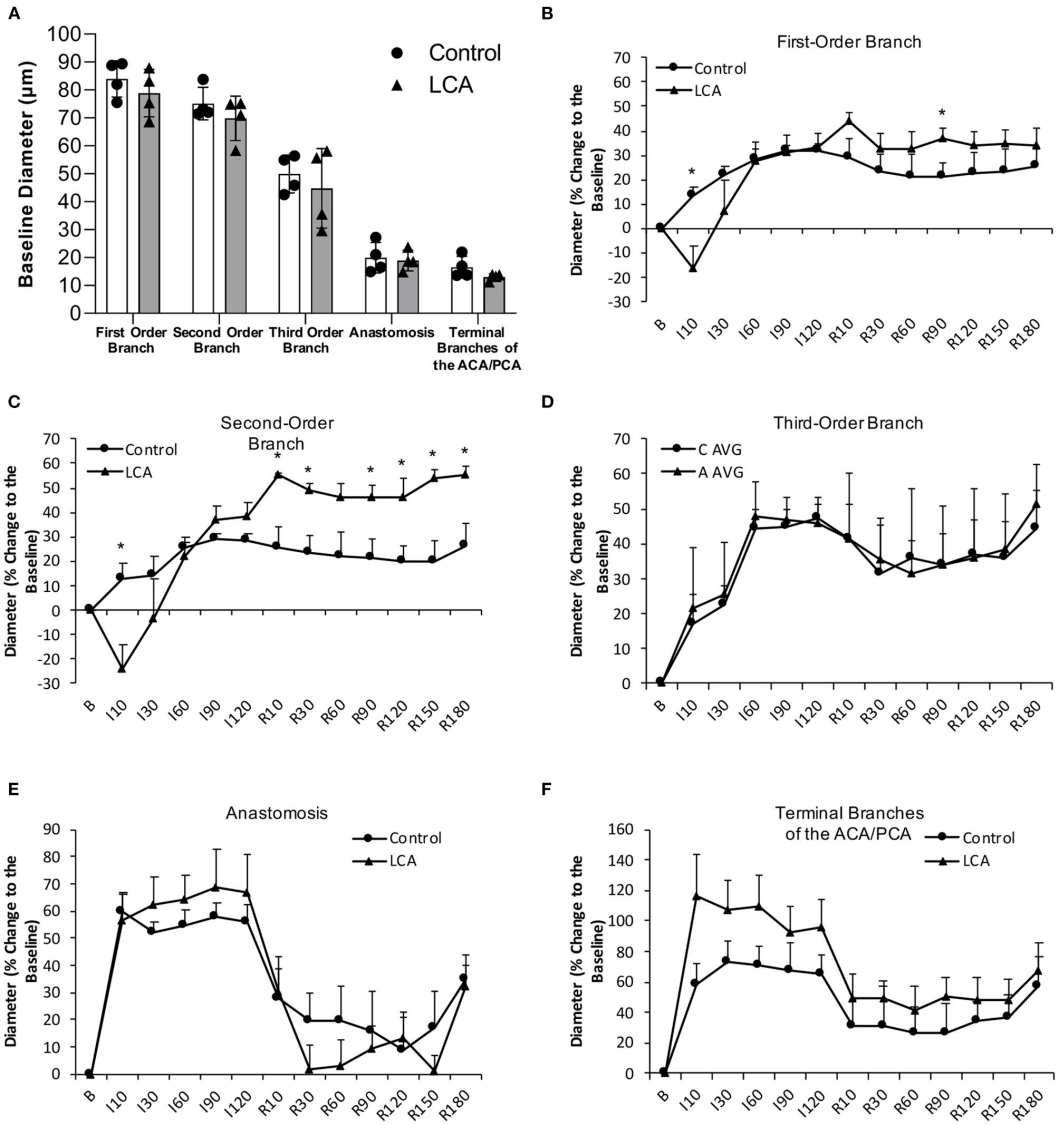
Effect of LAC on baseline diameter and vasoreactivity of the MCA, arterial anastomoses, and terminal branches of the ACA/PCA during 2-h MCA ligation and 3-h reperfusion. **(A)** Baseline diameter. Values are mean ± SD for four mice in each group. Analyzed using one-way ANOVA. **(B–D)** Vasoreactivity of the MCA. **(E)** Vasoreactivity of the arterial anastomoses. **(F)** Vasoreactivity of the terminal branches of the ACA/PCA. ^*^P < 0.05 vs. Control. Analyzed using unpaired *t*-test.

**Figure 6:**
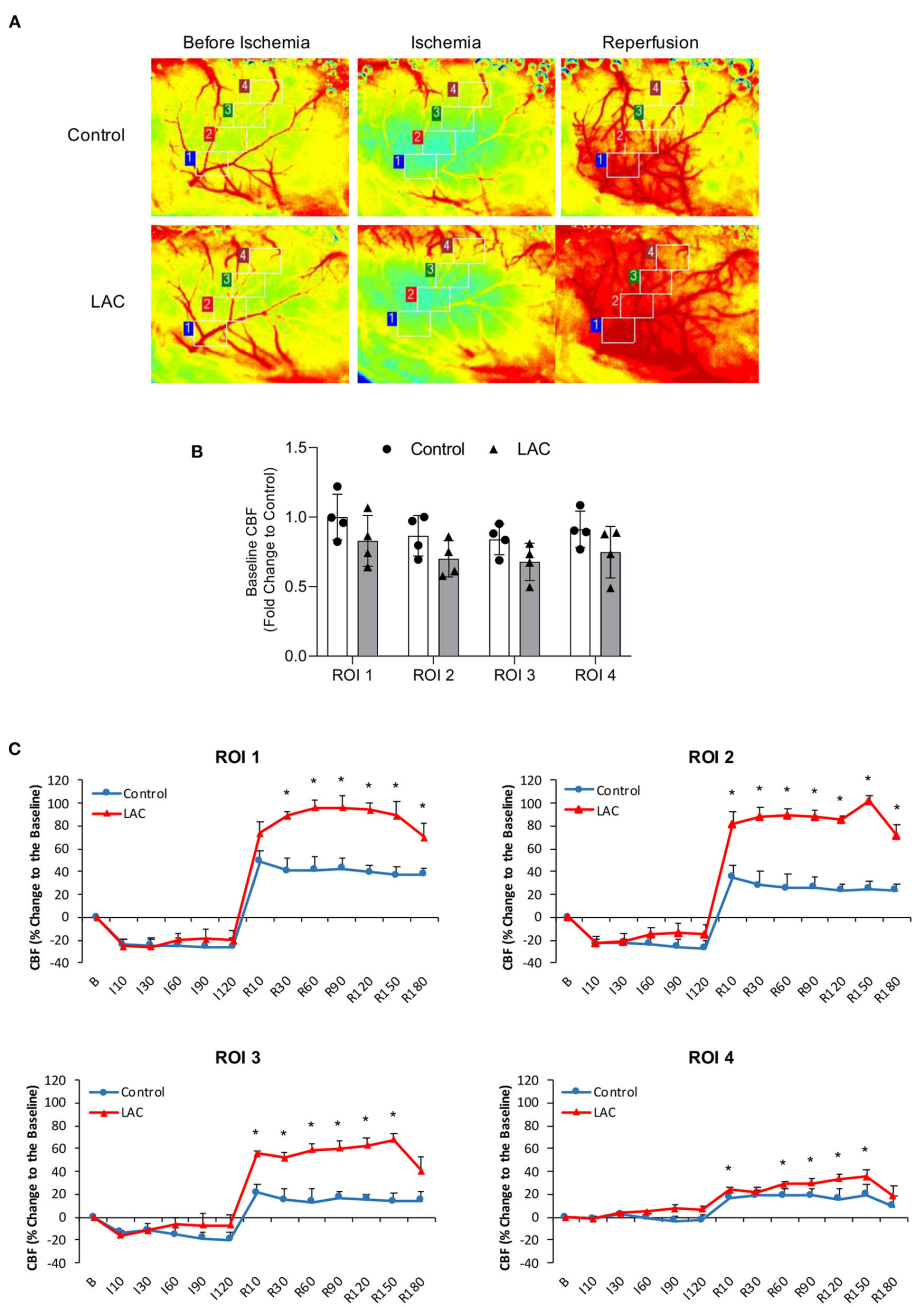
Effect of LAC on rCBF during 2-h MCA ligation and 3-h reperfusion. **(A)** Representative laser speckle images of rCBF. **(B)** Baseline rCBF. Values are mean ± SD for four mice in each group. Analyzed using one-way ANOVA. **(C)** Percentage changes of rCBF to the baseline during ischemia and reperfusion. ^*^P < 0.05 vs. Control. Analyzed using unpaired *t*-test.

There was no significant difference in baseline diameter of cortical branches of the MCA, arterial anastomoses, and terminal branches of the ACA/PCA between 0.7 g/kg/day ethanol-fed mice and control mice (Figure 5A). The ischemia (MCA ligation) produced vasodilation in all cortical branches of the MCA, arterial anastomoses, and terminal branches of the ACA/PCA during the entire 2-h ischemia (Figures 5B–F). The vasodilation of the first- and second-order branches of the MCA was significantly less at the beginning in 0.7 g/kg/day ethanol-fed mice compared to the control mice (Figures 5B,C). The vasodilation continued in all cortical branches of the MCA and terminal branches of the ACA/PCA during the entire 3-h reperfusion (release of the MCA ligation) (Figures 5B–F). The vasodilation of the second-order branch of the MCA was significantly greater in 0.7 g/kg/day ethanol-fed mice compared to the control mice (Figure 5C). On the other hand, there was no significant difference in baseline rCBF between 0.7 g/kg/day ethanol-fed mice and control mice (Figures 6A,B). The ischemia (MCA ligation) reduced rCBF in both groups. Although 0.7 g/kg/day ethanol feeding slightly increased rCBF in all four ROIs at the late stage of ischemia, it did not significantly alter the magnitude of reduction in rCBF during the entire 2-h ischemia (Figures 6A,C). Reperfusion (release of the MCA ligation) resulted in a hyperperfusion (hyperemia) in both groups during the entire 3-h reperfusion. However, the magnitude of hyperperfusion was significantly greater in 0.7 g/kg/day ethanol-fed mice compared to the control mice (Figures 6A,C).

## Discussion

In the present study, the influence of LAC and HAC on cerebral angiogenesis under physiological conditions and following transient focal cerebral ischemia was investigated. There are several new findings. First, both acute and chronic exposure to low-concentration alcohol increased angiogenic capability and upregulated VEGF-A and VEGFR2 in MBMVECs. Second, VEGFR2 inhibitor abolished acute low-concentration alcohol exposure-induced angiogenic capability. Third, LAC increased vessel density and number of branches in the cerebral cortex and subcortical area under physiological conditions and the peri-infarct cortex following transient focal cerebral ischemia. Fourth, LAC upregulated VEGF-A and VEGFR2 in the cerebral cortex under physiological conditions as well as following ischemic stroke. Fifth, LAC increased vasodilation and rCBF during reperfusion but not ischemia. Sixth, HAC had a minor inhibitory effect on cerebral angiogenesis and did not alter either VEGF-A or VEGFR2. We speculate that upregulation of VEGF-A and VEGFR2 may be involved in LAC-induced cerebral angiogenesis. In addition, the protective effect of LAC against cerebral I/R injury does not appear to be related to an alteration in cerebral vasoreactivity/rCBF during ischemia.

In our recent studies, two doses of ethanol altered cerebral I/R injury in mice. Eight-week gavage feeding with 0.7 g/kg/day ethanol before the ischemia significantly reduced infarct size and improved neurological function in a mouse model of transient focal cerebral ischemia. In contrast, 2.8 g/kg/day ethanol significantly increased infarct size and exacerbated neurological deficits (23, 24). The peak blood alcohol concentration was about 9 mM in 0.7 g/kg ethanol-fed mice and 37 mM in 2.8 g/kg ethanol-fed mice. Blood alcohol level at 9 mM is commonly seen following intake of one and a half American standard drinks (14 grams of ethanol/each), whereas blood alcohol concentration at 37 mM can be found following consumption of a little more than seven American standard drinks in a male with average body weight (70 kg) (26, 27). Thus, 0.7 and 2.8 g/kg/day ethanol in mice represent LAC and HAC in humans, respectively. The alcohol concentration declined rapidly and reached zero at 2 h after gavage feeding in 0.7 g/kg ethanol-fed mice but reduced slowly in 2.8 g/kg ethanol-fed mice (23). Thus, ethanol concentrations at 5, 10, and 50 mM were selected for our *in vitro* study. Two hours and 4 h were chosen as daily exposure times for chronic exposure of low-concentration and high-concentration ethanol, respectively.

A few studies have investigated the impact of alcohol on angiogenic capability in other types of endothelial cells. In an early study, Jones et al. reported that acute exposure of 1.0–2.5% ethanol increased tube formation in an endothelial-derived cell line (EA hy926) (28). Recently, Morrow et al. found that acute exposure of 10–50 mM ethanol increased tube formation in human umbilical vein endothelial cells (HUVECs). In addition, 25 mM ethanol increased proliferation and migration (29). In contrast, Radek et al. found that acute exposure to 100 mg/dl ethanol inhibits cord formation and tube formation in small-vessel murine endothelial cells 4-10 (SVEC4-10) (30). In the present study, 5 and 10 mM ethanol increased angiogenic capability when they were exposed to MBMVECs either acutely or chronically. However, 50 mM ethanol tended to increase angiogenic capability when it was acutely exposed but changed to decrease angiogenic capability when it was chronically exposed. The discrepancy between these studies regarding the acute effect of alcohol on angiogenic capability is not entirely clear but may be related to the timing and length of exposure to alcohol, the type of cell used, and/or species differences in the effects of alcohol. Previous studies also have investigated the influence of chronic alcohol consumption on angiogenesis in organs and tissues. A few studies found that chronic light-moderate alcohol consumption promoted tumor angiogenesis (31–33). In addition, chronic administration with moderate amounts of ethanol promoted angiogenesis in chick chorioallantoic membrane (CAM) (34). On the other hand, chronic prenatal administration of heavy alcohol impairs angiogenesis in the placenta and fetal brain (35). In a recent study, Zhang et al. found that LAC promoted but HAC attenuated ischemia-induced angiogenesis in the heart (10). In the present study, we found that LAC increased cerebral angiogenesis under basal conditions as well as following ischemic stroke. However, HAC tended to have little to no effect on cerebral angiogenesis. No studies that we are aware of have examined the influence of chronic alcohol consumption on cerebral angiogenesis. We speculate that the discrepancy regarding the effect of HAC on angiogenesis may be related to the dosage and length of alcohol administration. In addition, it is also possible that the organ systems are differently affected by alcohol. In the present study, LAC showed a regional difference in post-ischemic angiogenesis at 72 h of reperfusion. It increased vessel density by about 2-folds in the peri-infarct cortex but did not significantly increase vessel density in the peri-infarct subcortex. The mechanism underlying the difference is not clear. A previous study found that post-ischemic angiogenesis occurred earlier in the peri-infarct area than the infarct core (8). The infarct core induced by a 60-min MCAO mainly locates in the subcortex. Thus, the regional difference may be related to the location of the infarct core.

VEGF-A appears one of the most potent mediators in both physiological and pathological angiogenesis (12, 36). VEGF-A can bind with VEGFR1 and VEGFR2. However, VEGFR-1 may act as a decoy receptor, which competitively reduces the binding of VEGF-A to VEGFR-2 (12, 30). The activation of VEGFR-2 triggers a series of signaling events including PI3K/Akt, MEK/ERK, and MAPK pathways to promote survival, proliferation, and migration of endothelial cells. In the present study, we found that both VEGF-A and VEGFR2 were upregulated in low-concentration ethanol-exposed MBMVECs and the cerebral cortex of LAC mice. In addition, the upregulation appeared consistent with the increased angiogenic capabilities and cerebral angiogenesis. Previous studies have shown that VEGF plays an important role in the angiogenic activity of alcohol (10, 33, 34). In the present study, VEFGR2 expression was also upregulated by low concentration/low-dose alcohol. Moreover, the VEGFR2 inhibitor abolished alcohol-induced angiogenic capability. Thus, it is conceivable that LAC-induced cerebral angiogenesis may be related to the upregulation of VEGF-A and VEGFR2. However, it should be noted that VEGF-mediated VEGFR2 activation could increase BBB permeability *via* Src and PI3K/Akt pathways, especially following ischemic stroke (12). Interestingly, we recently found that post-ischemic BBB disruption was less in LAC mice compared to the control mice (23). LAC upregulated tissue inhibitor matrix metalloproteinase 1 (TIMP-1) and reduced MMP-9 activity in the cerebral cortex under physiological conditions. In addition, post-ischemic upregulation of IL-1beta was significantly less and post-ischemic upregulation of IL-1ra was significantly greater in LAC mice (23). Thus, although LAC upregulates VEGF-A and VEGFR2, it may prevent BBB disruption via its anti-inflammatory effect.

In the present study, we further determined the influence of LAC on regional vasoreactivity and rCBF before, during, and after ischemia. Unfortunately, LAC did not alter regional vasodilation during ischemia. In addition, rCBF during ischemia was not improved by LAC. Thus, it appears that although LAC promoted cerebral angiogenesis, it did not increase anastomotic connections at least between the MCA and the ACA/PCA. We speculated that the neuroprotective effect of LAC is not related to an alteration in cerebral vasoreactivity/rCBF during ischemia. In the present study, LAC significantly augmented the hyperemia during reperfusion. The mechanism underlying post-ischemic hyperemia is not completely clear. We previously found that antagonists of nitric oxide synthase (NOS) and cystathionine γ-lyase (CSE) significantly inhibited post-ischemic cerebral vasodilation/hyperemia (25, 37). Recently, CSE has been shown to be upregulated in the brain of LAC mice (38). Thus, the augmented post-ischemic hyperemia in LAC mice presumably resulted from the increased cerebral angiogenesis and upregulated CSE. It is not clear whether the increased hyperemia contributes to the neuroprotective effect of LAC. Post-ischemic hyperemia is a common phenomenon in both patients and animal models (39, 40). An early study reported that post-ischemic hyperemia was associated with the worst brain injury in patients with ischemic stroke (39). A recent study found that a larger infarct volume was observed in animals showing post-ischemic hyperemia following a 90-min focal cerebral ischemia (40). However, post-ischemic hyperemia usually was followed by normal perfusion and fast recovery of function when the duration of ischemia was short, whereas it was followed by hypoperfusion and impaired function when the duration of ischemia was prolonged (41). We previously found that endogenous vasodilators including NO and H_2_S but not the hyperemia *per se* were involved in post-ischemic BBB disruption. The post-ischemic BBB disruption was significantly reduced when the hyperemia was preserved (25). Moreover, pre-ischemic angiogenesis reduced brain injury and improved functional outcomes following transient focal cerebral ischemia (2). Thus, we speculate that the impact of post-ischemic hyperemia on ischemic brain damage is dependent on the length of ischemia and the mechanism of the hyperemia. The impact of LAC-induced cerebral angiogenesis on ischemic stroke may need to be further determined with different lengths of ischemia.

We know of no studies that have examined the mechanism underlying alcohol-induced upregulation of VEGF-A and VEGFR2. Recently, we found that LAC significantly increased nuclear PPARγ protein/DNA binding activity in the cerebral cortex (42, 43). Moreover, PPARγ was upregulated in low-concentration alcohol-exposed endothelial cells and neurons (42, 43). PPARγ is a ligand-activated transcription factor expressed in the central nervous system and vascular endothelial cells. A previous study found that PPARγ activation upregulated VEGF-A and VEGFR2 and increased angiogenic capability in cardiac myofibroblasts (44). In addition, transfection of the PPARγ gene increased the promoter activity of VEGF-A (45). Thus, it is possible that LAC upregulates VEGF-A and/or VEGFR2 by activating PPARγ. On the other hand, VEGF-A is one of the primary target genes regulated by hypoxia-inducible factor (HIF), which is a major mediator of hypoxic response (46). Acute high-dose alcohol intake upregulated HIF-1α in a rat model of traumatic brain injury (47). In addition, Reddy et al. found that chronic alcohol consumption upregulated HIF-1α in the cerebral cortex of adult rats (48). Thus, it is also possible that HIF-1α is involved in the LAC-induced upregulation of VEGF-A. Future studies will determine the roles of PPARγ and HIF in alcohol-induced cerebral angiogenesis. In addition to VEGF-A/VEGFR2 signaling, a few studies reported that Notch, angiopoietin-1/Tie2, and VEGFR1/Flt-1 pathways may contribute to alcohol-induced angiogenesis (29, 31, 34). It would be interesting to determine their role in alcohol-induced cerebral angiogenesis in the future. Interestingly, alcohol consumption also dose-dependently affects cognitive function. Light to moderate alcohol use appears to associate with a decreased risk of cognitive impairment and dementia (49, 50). It is not clear whether LAC-induced cerebral angiogenesis associate with reduced cognitive impairment. A future study determining the influence of LAC on CBF responses to stimuli appears needed.

In summary, the present study was the first to determine the influences of alcohol on cerebral angiogenesis. We found that HAC had a mild inhibitory effect on cerebral angiogenesis, whereas LAC promoted cerebral angiogenesis under physiological conditions and following transient focal cerebral ischemia. Furthermore, LAC-induced cerebral angiogenesis failed to improve rCBF during ischemia but increased rCBF during reperfusion. A future study determining the mechanism of LAC-induced upregulation of VEGF-A and VEGFR2 is needed. Understanding how alcohol affects ischemic stroke will not only advance clinical management of ischemic stroke in alcohol users but also may lead to novel strategies to prevent and treat ischemic stroke in non-alcohol users with a high risk of developing ischemic stroke.

## Data Availability Statement

The raw data supporting the conclusions of this article will be made available by the authors, without undue reservation.

## Ethics Statement

The animal study was reviewed and approved by the Institutional Animal Care and Use Committee (IACUC) at the Louisiana State University Health Science Center (LSUHSC)-Shreveport.

## Author Contributions

HS conceived the experiments. JL, CL, EL, SM, MP, XL, and HS conducted the experiments. JL, CL, and HS analyzed the results. JL wrote the main manuscript text and prepared all of the figures. All authors reviewed the manuscript.

## Funding

This study was supported by a National Institutes of Health (NIH) Grant (AA023610) and funds from Louisiana State University Health Sciences Center-Shreveport to HS, and a Malcolm Feist Predoctoral fellowship to JL from the Center of Excellence for Cardiovascular Diseases and Sciences, Louisiana State University Health Science Center-Shreveport.

## Conflict of Interest

The authors declare that the research was conducted in the absence of any commercial or financial relationships that could be construed as a potential conflict of interest.

## Publisher's Note

All claims expressed in this article are solely those of the authors and do not necessarily represent those of their affiliated organizations, or those of the publisher, the editors and the reviewers. Any product that may be evaluated in this article, or claim that may be made by its manufacturer, is not guaranteed or endorsed by the publisher.

## Abbreviations

ACA: anterior cerebral artery
BBB: blood-brain barrier
bFGF: basic fibroblast growth factor
CCA: common carotid artery
CSF: cerebrospinal fluid
ECA: external carotid artery
HAC: heavy alcohol consumption
HIF: hypoxia inducible factor
ICA: internal carotid artery
LAC: light alcohol consumption
MABP: mean arterial blood pressure
MBMVECs: mouse brain microvascular endothelial cells
MCA: middle cerebral artery
MMPs: matrix metalloproteinases
PCA: posterior cerebral artery
PDGF: platelet-derived growth factor
rCBF: regional cerebral blood flow
TGFβ: transforming growth factor beta
VEGF-A: vascular endothelial growth factor A
VEGFR2: vascular endothelial growth factor receptor 2.
