# Supplementary material for: Chronic Low-Dose Alcohol Consumption Promotes Cerebral Angiogenesis in Mice
Source: Front Cardiovasc Med. 2021 Nov 17;8:681627. doi: 10.3389/fcvm.2021.681627 (PMC8635527; doi:10.3389/fcvm.2021.681627)

Original Western Blots

Figure 1D

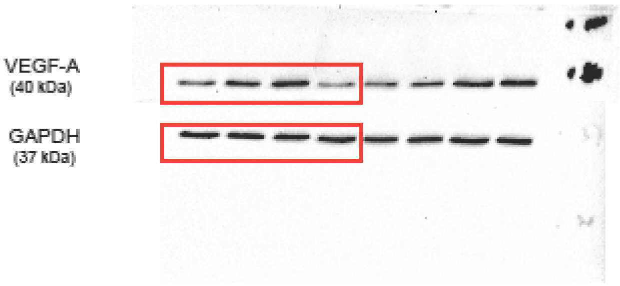

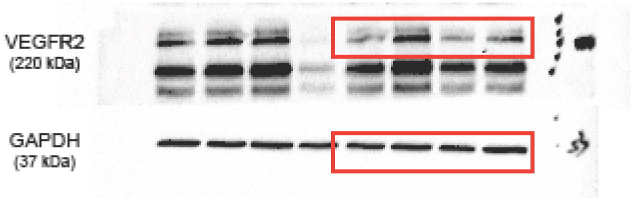

From the original experiment

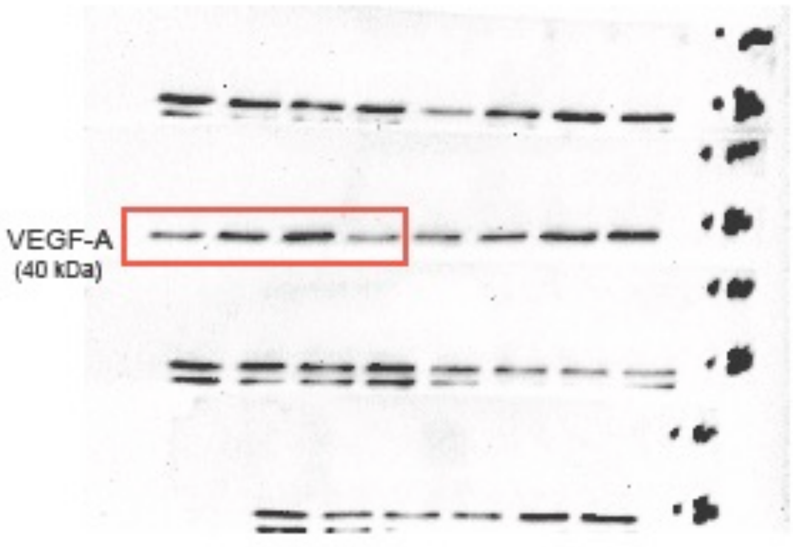

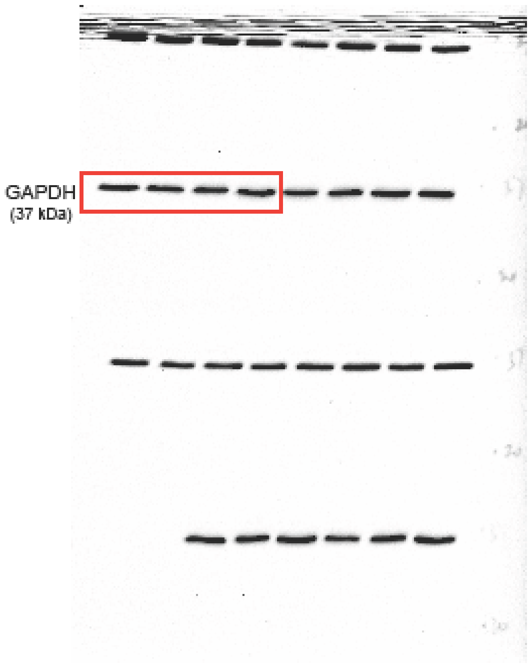
GAPDH
(37 kDa)

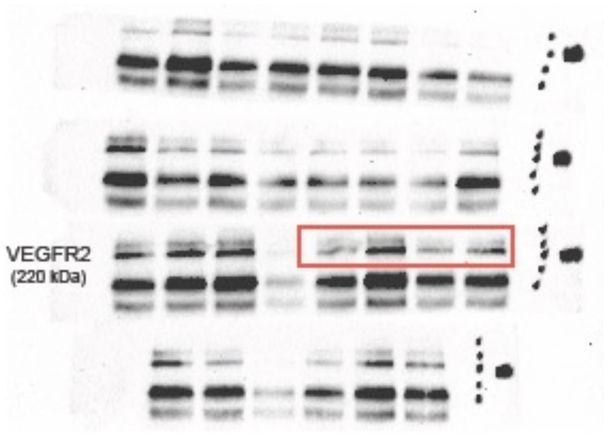
VEGFR2
(220 kDa)

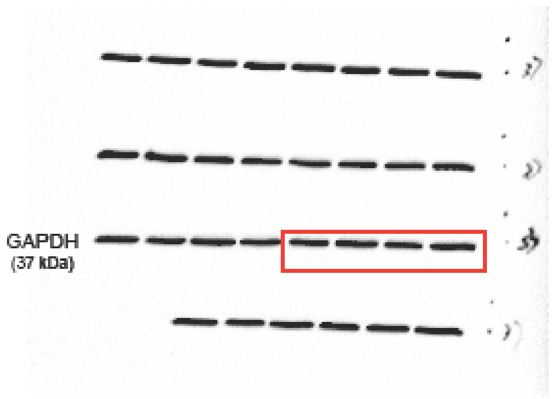
GAPDH
(37 kDa)

Figure 2D

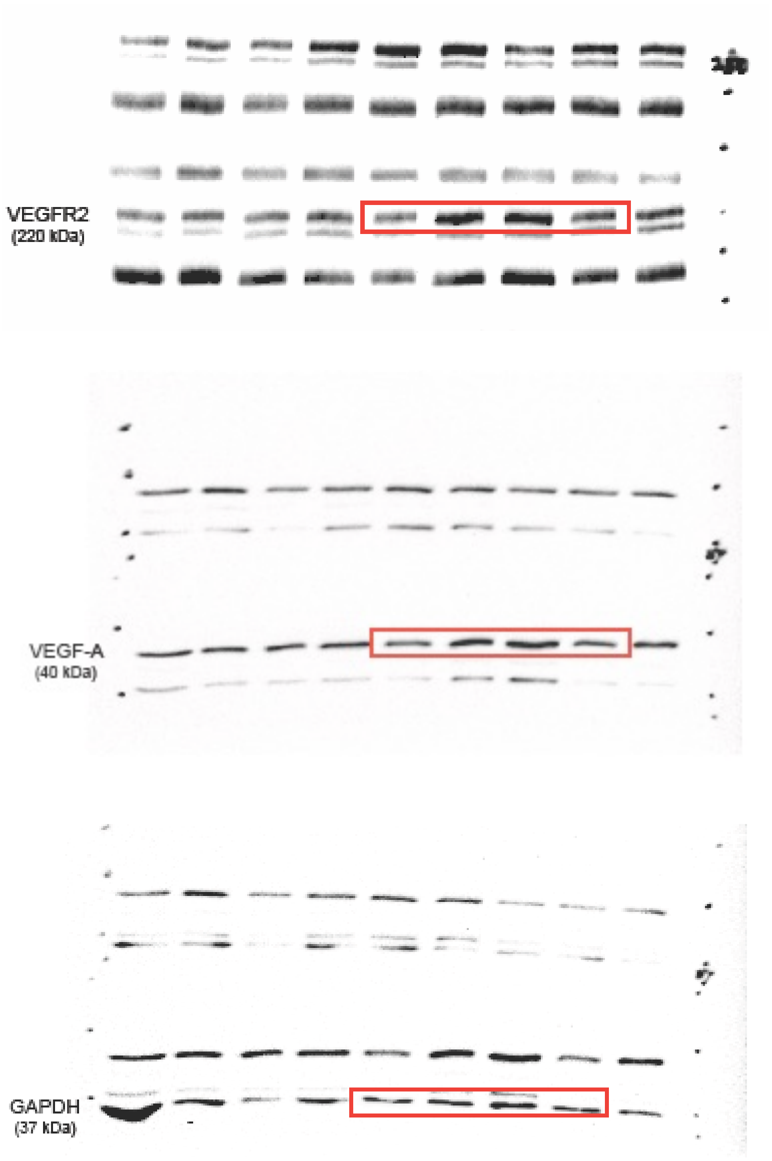

Following longer exposure time

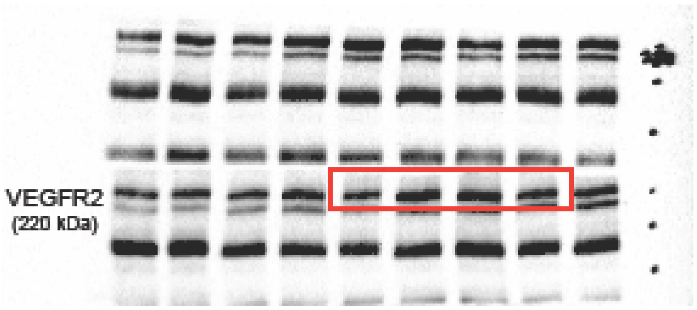

VEGF-A
(40 kDa)

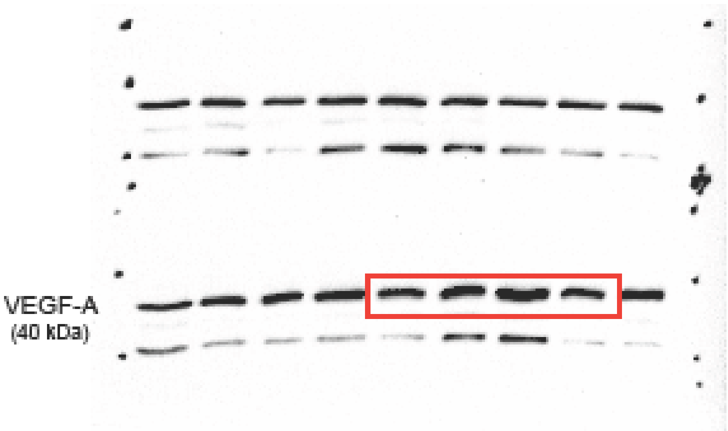

GAPDH
(37 kDa)

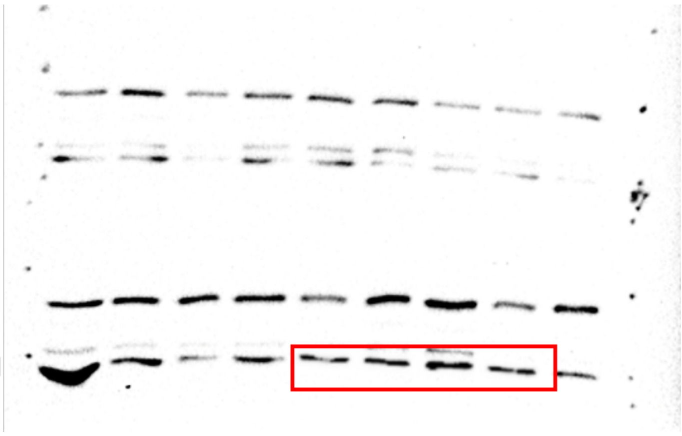

Figure 3C

VEGF-A
(40 kDa)

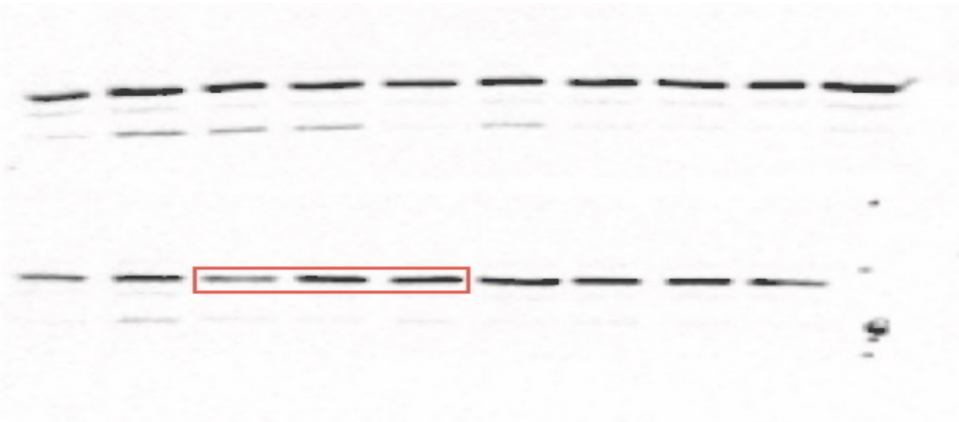

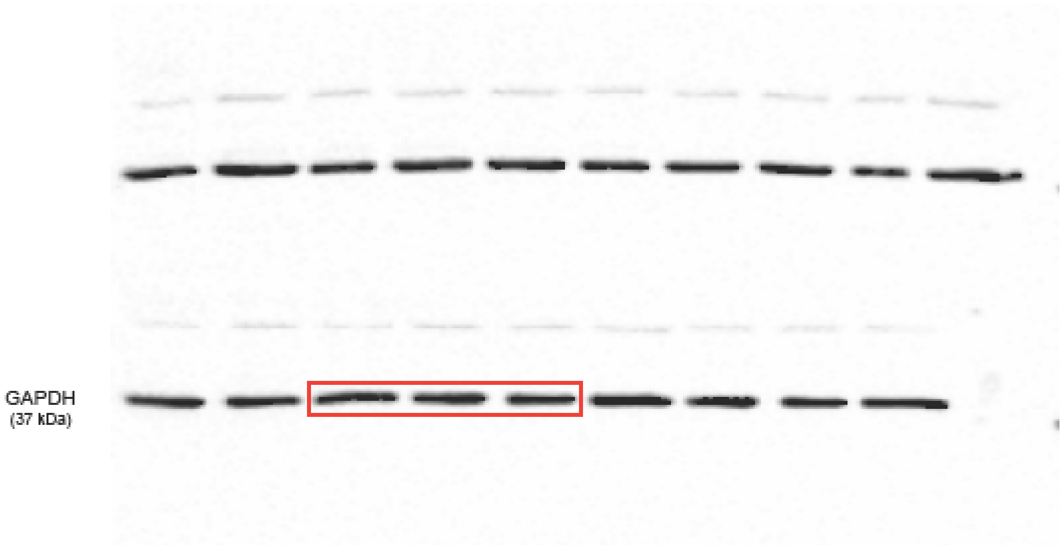
GAPDH
(37 kDa)

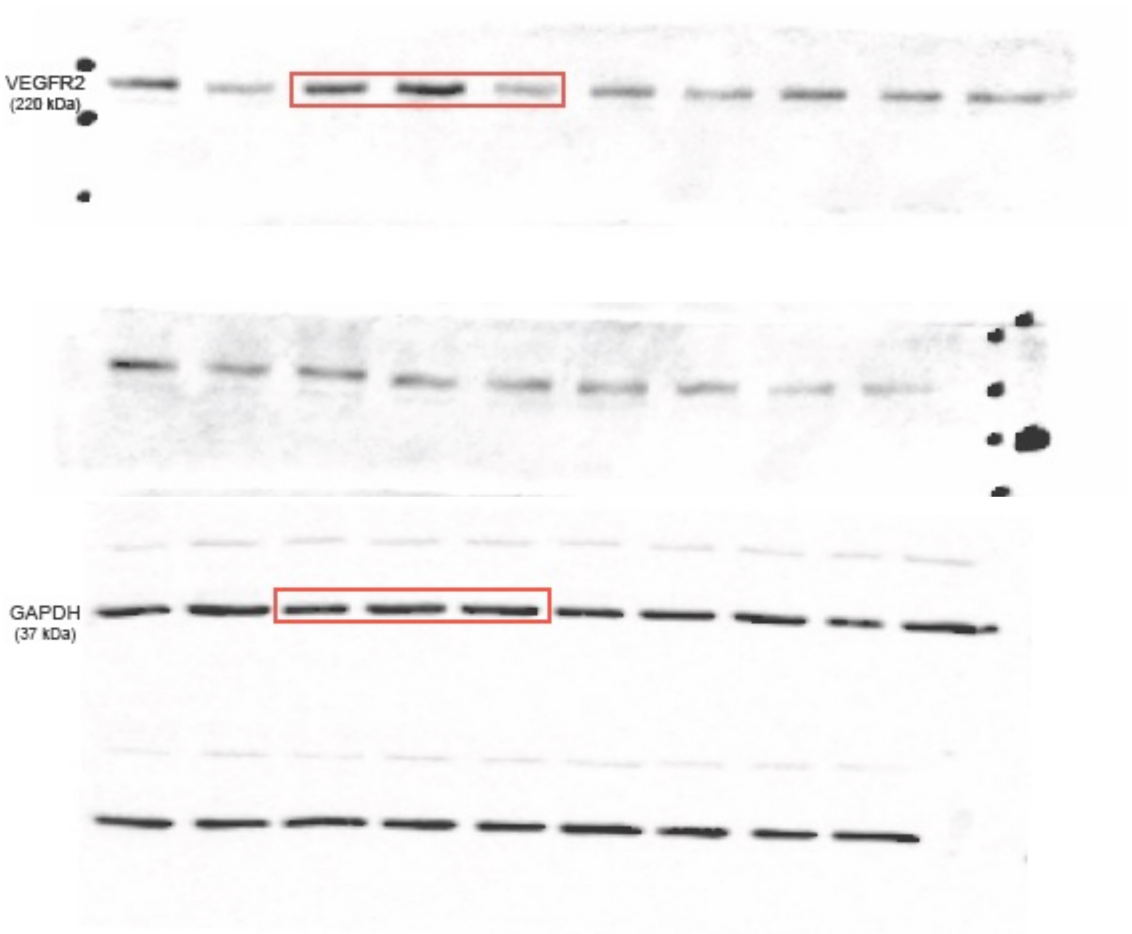
VEGFR2
(220 kDa)
GAPDH
(37 kDa)

Following longer exposure time

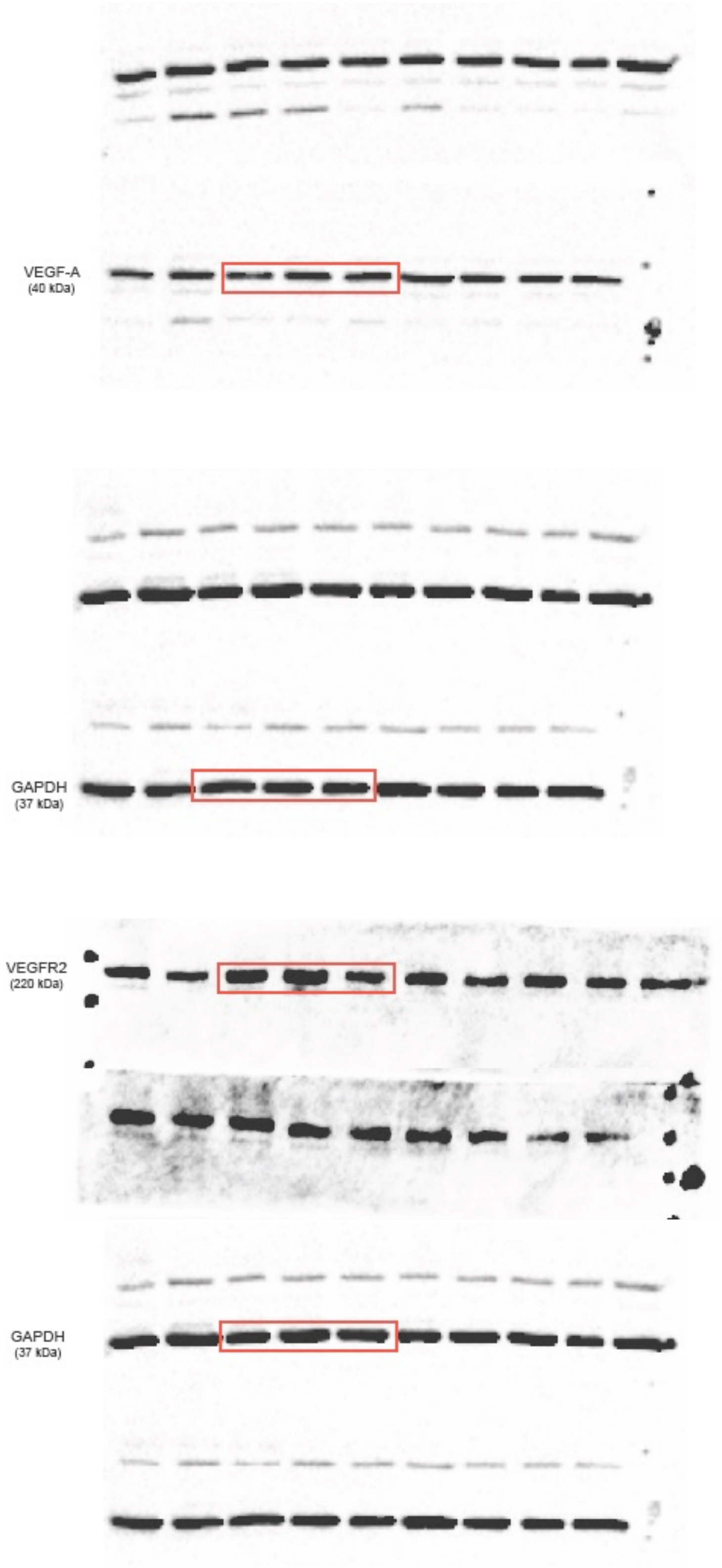

Figure 4C

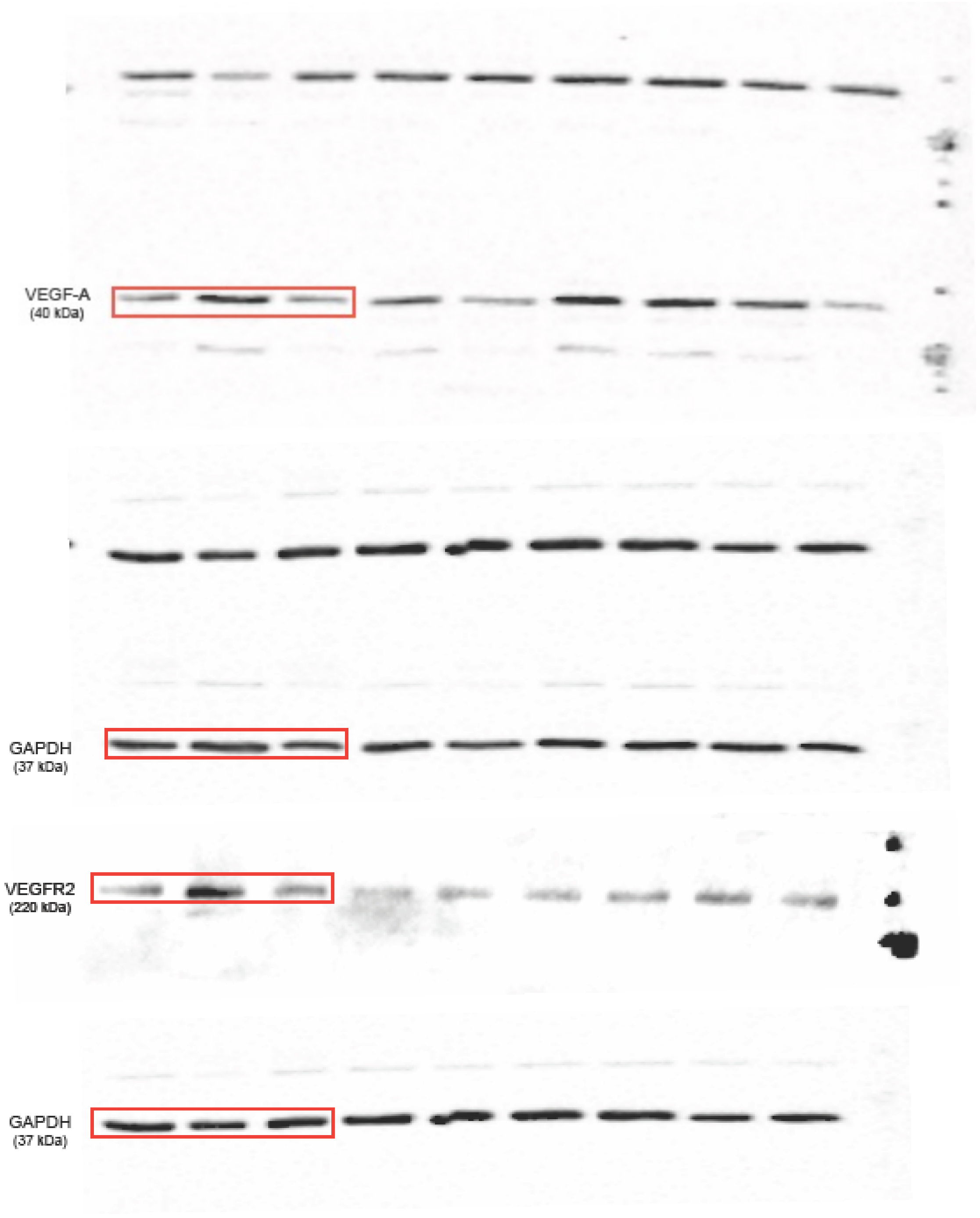

Following longer exposure time

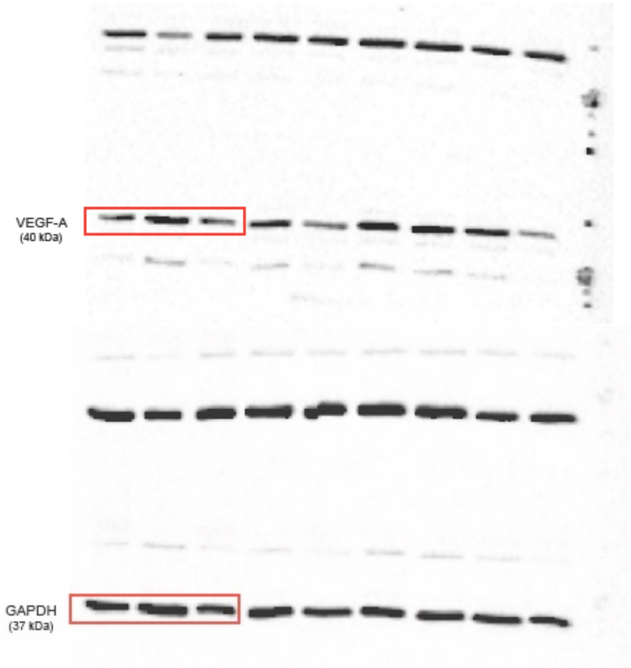

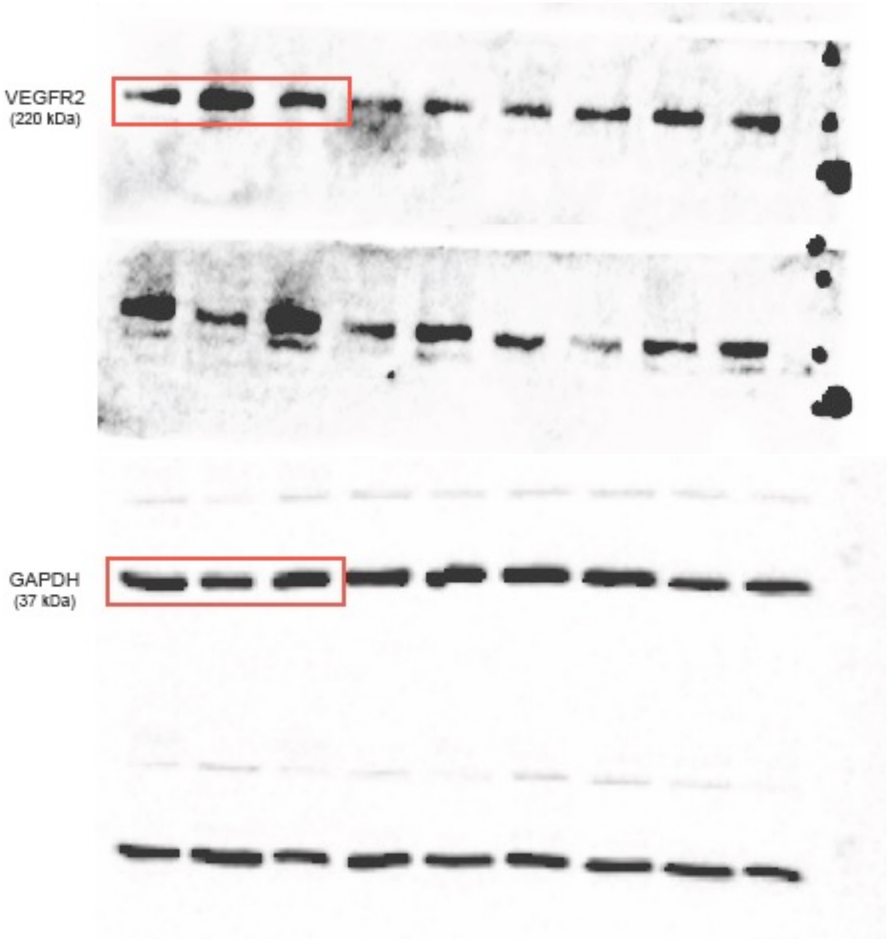

Supplement: Supplementary file 1 [file Data_Sheet_1.PDF]
